# Management of asymptomatic hypoglycemia with 40% oral dextrose gel in near term at-risk infants to reduce intensive care need and promote breastfeeding

**DOI:** 10.1186/s13052-021-01149-7

**Published:** 2021-10-09

**Authors:** Fabio Meneghin, Martina Manzalini, Miriam Acunzo, Irene Daniele, Petrina Bastrenta, Francesca Castoldi, Francesco Cavigioli, Gian Vincenzo Zuccotti, Gianluca Lista

**Affiliations:** 1 NICU, Department of Pediatrics, V. Buzzi Children’s Hospital, ASST-FBF-Sacco, Milan, Italy; 2 Department of Pediatrics, University of Milan, V. Buzzi Children’s Hospital, ASST-FBF-Sacco, Milan, Italy

**Keywords:** Hypoglycemia, At-risk newborns, Breastfeeding, 40% oral dextrose gel, Neonatal intensive care unit, NICU admission

## Abstract

**Background:**

Neonatal hypoglycemia is a common disorder especially in at-risk infants and it can be associated with poor long-term neurological outcomes. Several therapeutic interventions are suggested, from the implementation of breastfeeding to the glucose intravenous administration. Oral dextrose gel massaged into the infant’s inner cheek is a recent treatment option of asymptomatic hypoglycemia, after which oral feeding is encouraged. This approach seems to reduce the admission of infants to neonatal intensive care unit (NICU) so favouring maternal bonding and breastfeeding success at discharge.

**Methods:**

In our ward, we prospectively compared a group of near-term neonates, (Gr2, *n* = 308) at risk for hypoglycemia, treated with an innovative protocol based on the addition of 40% oral dextrose gel (Destrogel, Orsana®,Italy) administered by massaging gums and cheek with historical matching newborns (Gr1, *n* = 389) treated with a formerly used protocol, as control group. The primary outcome was occurrence of NICU admission and the requirement of intravenous glucose administration; while discharge with full breastfeeding was the secondary outcome.

**Results:**

In Gr1, 39/389 (10%) infants presented with asymptomatic hypoglycemia, 19/39 were transferred to the NICU, and 14/39 required intravenous glucose treatment. In Gr2, among the 30/308 infants with asymptomatic hypoglycemia managed according to the new protocol, 3/30 were transferred to the NICU and received intravenous glucose infusion. The mean duration of hospitalization respectively was 6.43 (± 6.36) and 3.73 ± 1.53 days (*p* <  0.001). At discharge, 7.7% of the infants in Gr1 and 30% of the infants in Gr2 were exclusively breastfed (*p* = 0.02). Considering Gr1 vs Gr2, the number of patients that were transferred to NICU was 19 (48.7%) vs 3 (10%) (*p* = 0.001) and the number of infants that needed intravenous glucose infusion was 14 (35.9%) vs 3 (10%) (*p* = 0.01), respectively.

**Conclusions:**

In our population of near term infants, the introduction of 40% oral dextrose gel to the protocol, helped in the safe management of asymptomatic hypoglycemia and, at the same time, implemented breastfeeding.

## Background

Hypoglycemia is the most frequent metabolic disorder in neonates, with an incidence of 5–15% in healthy term infants and up to 50% in infants with risk factors [1]. During the pregnancy the fetus receives glucose from placental circulation, but at birth this supply stops abruptly and the neonates need to become independent to produce energy. In the first 2 hours of life, glycemia reaches the lowest level, then the values stabilize between 4 and 6 h of life [2].

Neonatal hypoglycemia, especially if prolonged, can be associated with brain injury and poor neurodevelopment outcomes, including cognitive impairment, sensor disability, cerebral palsy, seizures and developmental delay [3, 4]. Despite this, uncertainty persists regarding the definition of neonatal hypoglycemia and about the correlation between the values of glycemia, the symptoms in newborns and the long-term sequaelae [5, 6]. Moreover, even the inaccuracy of the measurement tools can complicate the interpretation of the data [7]. The two most recent international guidelines from the Pediatric Endocrine Society and the American Academy of Pediatrics do not help to clarify the correct values to define hypoglycemia in neonates [8, 9].

Treatment options vary according to the single cases, the occurrence of symptoms and of blood glucose levels. The first intervention is early and frequent oral feeding, focused on breastfeeding, and infant formula supplementation can be provided if human milk is not available. Failing this approach, infants who remain hypoglycemic are often transferred in neonatal intensive care unit (NICU) or specialty care nursery and intravenous (IV) glucose is administered. NICU admission leads to physical separation between mothers and neonates, causing negative impact on bonding and to a delayed establishment of breastfeeding. More recently, the buccal administration of dextrose gel showed positive effects on reducing the time of mother-infant separation and increasing the likelihood of breastfeeding at discharge [10]. By the direct application to the oral mucosa, glucose can rapidly enter the systemic circulation via the lingual and internal jugular veins, even if a rate of the dose may also be swallowed and absorbed by the gastrointestinal tract [11].

For these reasons, in late 2019, we decided to implement a protocol for the management of hypoglycemia in at-risk newborns in our Neonatal Unit including a commercially sourced 40% oral dextrose gel (Destrogel, Orsana®, Italy). After this, we conducted a comparison on the impact of intensive care need (NICU admission and intravenous glucose administration) and breastfeeding success at discharge between a population of near term at-risk infants for asymptomatic hypoglycemia managed with 40% oral dextrose gel and a historical group.

## Methods

A retrospective group (Gr1) of near-term neonates at risk for hypoglycemia, managed during the first 24 h of life with a specific protocol at V. Buzzi Children’s Hospital (ASST-FBF-Sacco, Milan, Italy) enrolled between May 2019 and October 2019, was compared with a prospective group (Gr2) managed at the same institution with a new protocol starting in November 2019.

Neonates in group 2 (Gr2) were enrolled between November 2019 and April 2020 and the new protocol introduced the use of 40% oral dextrose gel (Destrogel, Orsana®,Italy) administered by massaging between the gums and cheek.

For both groups the following criteria were considered risk factors for hypoglycemia: mild prematurity (> 35 weeks’ gestational age GA), post-term birth (> 41 + 6 weeks’ gestational age GA), low birth weight (LBW; < 10° percentile), large for gestational age (LGA, > 90° percentile), intrauterine growth restriction (IUGR), birth weight under 2500 g or above 4000 g, maternal diabetes, transient mild neonatal respiratory distress, urgent cesarean section for foetal distress, maternal eclampsia and hypertension, meconium-stained amniotic fluid, foetal eritroblastosis, polycythemia, mild hypothermia, metabolic acidosis at birth, maternal pharmacological therapy during pregnancy with β-blockers, β-sympathomimetics or oral hypoglycemic agents, congenital syndromes and twins with discordant neonatal birth weight more than 10%.

We excluded infants born before 35 weeks’ GA, and newborns with symptomatic hypoglycemia.

The study was approved by our local IRB (Institutional Review Board) and parental consents were obtained. A complete database with all newborn characteristics was drafted.

In both study groups, skin-to-skin contact was promoted at birth, the first meal within the first hour of life was ensured (breastfeeding if possible, otherwise squeezed human milk or formula), and glycemic monitoring was initiated 30 min after the first meal. Monitoring duration varied according to risk factor (24 h in case neonates are SGA, IUGR, birth weight < 2500 g, preterm; 12 h if LGA neonates or maternal diabetes, maternal preeclampsia/eclampsia or hypertension, use of medications in pregnancy, such as beta blockers, oral hypoglycemic agents, intrapartum maternal glucose infusion).

The main difference between the two protocols is that in the old one, at the first finding of glycaemia < 25 mg/dl, if confirmed by blood gas analysis in NICU, the newborn was placed in infusion, while in the following protocol the neonate always received oral dextrose gel. In both protocols, however, two consecutive findings of glycemia < 25 mg/dl were an absolute indication to IV glucose therapy.

### Group 1 (retrospective)

**Fig. 1:**
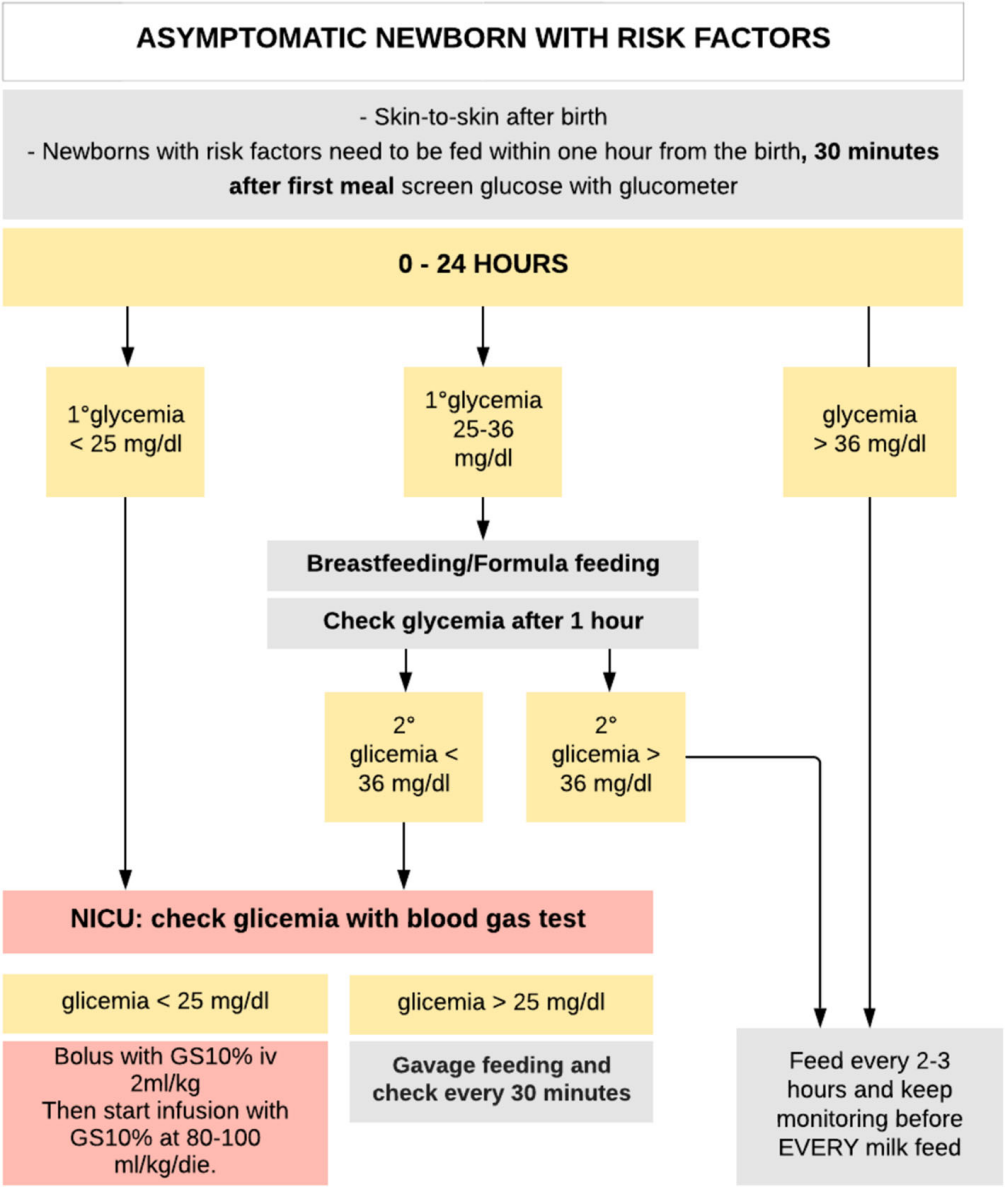
Group 1 flow chart

At the first glycemic checkup, if glycemia was < 25 mg/dl the newborn was transferred directly to NICU, while if glycemia ranged between 25 and 36 mg/dl the newborn was fed (breastfeeding, squeezed human milk or formula milk) and only after a further blood glucose below 36 mg/dl, the newborn was taken to the NICU. In NICU a blood gas analysis was performed: if blood glucose was < 25 mg/dl then IV glucose was administered, while if blood glucose was between 25 mg/dl and 36 mg/dl a squeezed human milk or artificial milk was initiated by gavage (see Fig. 1).

### Group 2 (prospective)

#### 0–4 H of life

First glycemic control: if glycemia was lower than 40 mg/dl, 40% oral dextrose gel at 200 mg/kg was administered followed by breast-feeding, squeezed human milk or artificial milk.

Second glycemic check: if glycemia ranged from 25 mg/dl to 40 mg/dl, it was possible to repeat the administration of oral dextrose gel; if glycemia was lower than 25 mg/dl, the newborn was transferred to the NICU to start the administration of intravenous glucose.

#### 4–24 h of life

**Fig. 2:**
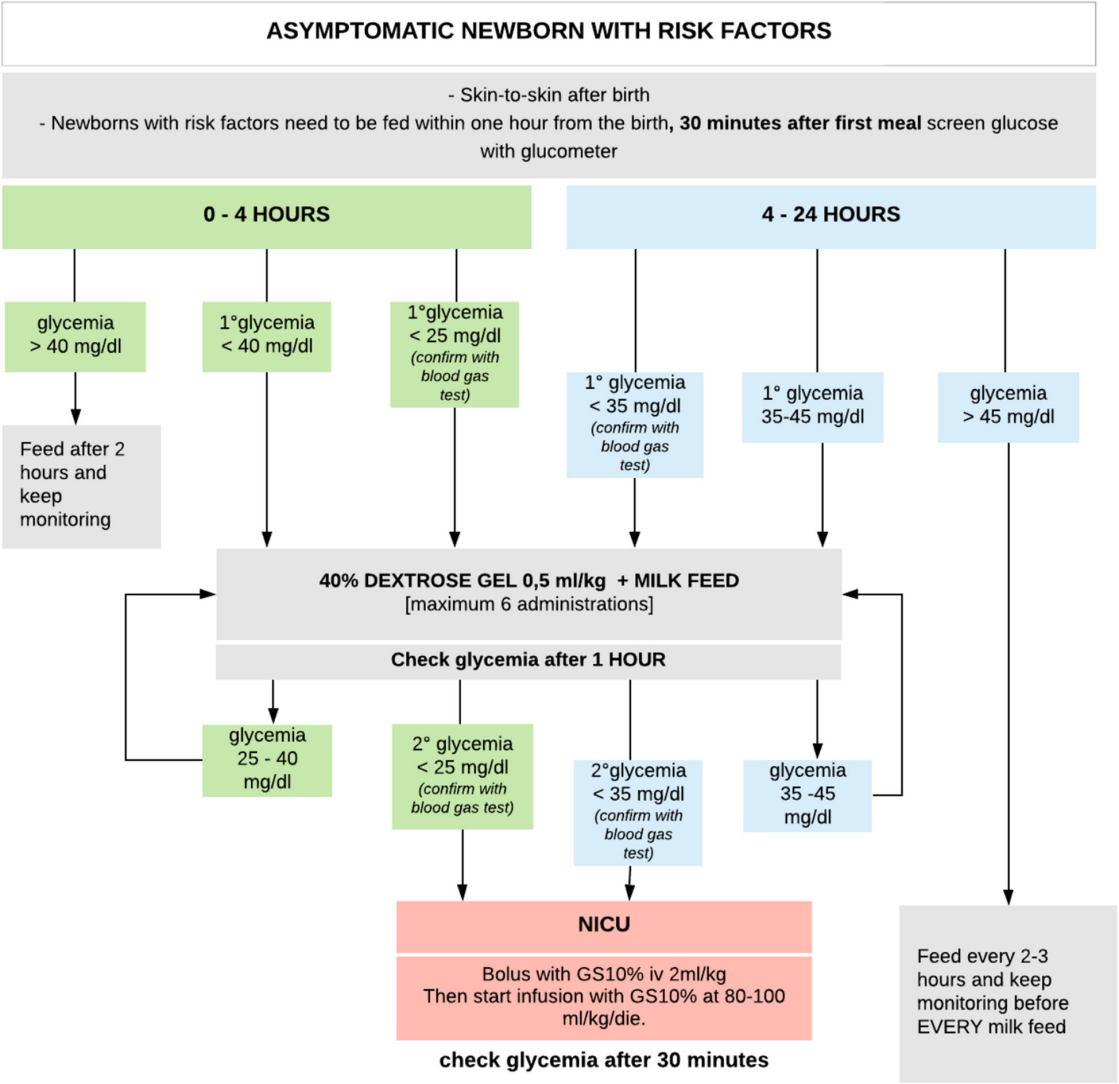
Group 2 flow chart

When the first glycaemia from 4 h of life was resulted lower than 45 mg/dl, oral dextrose gel was administered, followed by breast-feeding, squeezed human milk or artificial milk. Dextrose gel can be administered up to 6 times if blood glucose levels ranged between 35 mg/dl to 45 mg/dl at subsequent controls. If, instead, starting from the second checkup the glycemia was lower than 35 mg/dl, the newborn was brought to the NICU to undertake the administration of intravenous glucose (see Fig. 2).

The primary outcome of the study was comparing rates of access to the NICU and the need for IV glucose therapy. Secondary outcomes were the rate of exclusive breastfeeding at discharge (defined as the newborn receiving only breast milk the last feeding before discharge) and the length of hospitalization.

A statistical analysis was performed: a t-Student test was provided to analyze continuous variables, while for categorical variables like transfer to NICU, need for intravenous treatment and the rate of exclusive breastfeeding at discharge a χ^2^ test was used. Statistical significance was established for values of *p* <  0.05. The SPSS software was used to analyze data.

## Results

The neonatal baseline characteristics of the two groups are shown in Table 1.

**Table 1:** Baseline Characteristic

	Gr1 retrospective	Gr2 prospective	***P***-value
Number	389	308	
Males	215 (55.3%)	181 (58.8%)	0.86
Birthweight (g)	3166	3291	0.017
Gestation (wks)	38 + 5	39 + 1	0.002
Apgar score < 5 at 5 min	0	0	
Vaginal birth	277 (71.2%)	227 (73.7%)	0.53
pH at birth	7,29	7,27	0.02

The risk factors for hypoglycemia in the two study groups were similar [Table 2].

**Table 2:** Risk factors for hypoglycemia

	Gr1	Gr2
Maternal diabetes	128 (32.9%)	94 (30,5%)
LBW	96 (24.7%)	79 (26.7%)
Birth weight > 4000 g	54 (13.9%)	50 (16,2%)
Prematurity	73 (18.8%)	36 (11,2%)
LGA	36 (9,2%)	35 (11,4%)
Perinatal distress	0	4 (1,3%)
IUGR	2 (0,5%)	3 (1%)
Hypothermic infants	0	2 (0,7%)
Maternal drugs	0	1 (0,5%)

Among infants in Gr1: 39/389 (10%) of the infants presented asymptomatic hypoglycemia. Of these, 19/39 (48.7%) were transferred to the NICU and 14/39 (35.9%) required intravenous glucose treatment. In 5/19 infants transferred to the NICU with hypoglycemia, the blood gas analysis did not confirm < 25 mg/dl and were managed with gavage with squeezed human milk or artificial milk. The mean length of hospitalization was 6.43 (± 6.36) days for a total of 248 days. At discharge 3 (7.7%) asymptomatic hypoglycemic infants were exclusively breastfed, and 1/3 received a complementary feeding during hospital stay.

Among infants in Gr2: 37/308 (12%) of the infants presented asymptomatic hypoglycemia. 7 infants were excluded from the study because of protocol deviations (non-use of oral dextrose gel). 3/30 (10%) were transferred to the NICU and all received intravenous glucose infusion. The mean length of hospitalization was 3.73 (± 1.53) days for a total of 133. At discharge 9/30 (30%) of newborns were discharged with exclusive breastfeeding.

The number of NICU admission and of glucose treatment, were significantly lower in Gr2 than that in the Gr 1. Moreover, the length of hospitalization in the Gr2 was significantly shorter than that in the first one and the number of exclusively breastfed infants was greater in the second group when compared with the first one (*p* < 0.001 and 0,02, respectively), as shown in Table 3.

**Table 3:** Comparison of outcomes in the two groups

	Gr1 ***n*** = 39	Gr2 ***n*** = 30	***P***-value
NICU admission (n,%)	19 (48.7%)	3 (10%)	0.001
Glucose intravenous therapy (n,%)	14 (35.9%)	3 (10%)	0.01
Length of hospitalization (mean ± SD)	6.43 ± 6.36	3.73 ± 1.53	< 0.001
Exclusive Breastfeeding at discharge (n,%)	3 (7.7%)	9 (30%)	0.02

## Discussion

Neonatal hypoglycemia still remains a challenge, due to the uncertainty in its definition and in the threshold to consider intervention [12]. For asymptomatic at-risk neonates, management is focused on normalizing their blood glucose levels and preventing both short and long-term severe neurological sequelae. Secondary but relevant target is to reduce physical separation between mothers and newborns so enhancing bonding and breastfeeding success. The introduction of the administration of 40% oral dextrose gel in the protocols for hypoglycemia was targeted to control neonatal asymptomatic hypoglycemia until feeding was established, to reduce the need of glucose intravenous therapy and NICU admission, and to promote breastfeeding and maternal bonding.

We analyzed the data from two groups of newborns at-risk for hypoglycemia, managed with (Gr2, prospective study group) or without (Gr1, retrospective control group) the administration of 40% oral dextrose gel.

In the last decades, the incidence of neonatal hypoglycemia in otherwise healthy infants is 5–15% [1, 13]; in our experience this data is respected, because we found an incidence of 10% (39/389) in the historical group (Gr1) and 12% (37/308) in the cohort of infants managed with the new protocol (Gr2).

Gr2 presented a reduced need of NICU admission, of IV therapy and a shorter length of hospitalization. Moreover, the rate of exclusively breastfed newborns at discharge, was higher in this group of infants when compared to the infants of the historical group (Gr1).

We found a significative different incidence of NICU admission for hypoglycemia: 48.7% (19/39) in the historical cohort of patients (Gr1) versus 10% (3/30) in the cohort of patients treated with 40% oral dextrose gel (Gr2). This finding is similar to the data reported in the literature. Rawat et al. [14] demonstrated that the introduction of oral dextrose gel for the management of hypoglycemia reduced the hospitalization rate from 42 to 26% (*p* < 0.01). Similar findings were found by Scheans et al. [15], who demonstrated that in the first year of use of dextrose gel the admission at NICU due to hypoglycemia was reduced of 73%. In other two studies performed in Australia [16] and USA [17], the Authors found a reduction of admission to NICU for the treatment of hypoglycemia of 15 and 7.7%, respectively. Rawat et al. [14] demonstrated a reduction of IV therapy of 15.5%, Gregory et al. [18] showed a reduction from 8.6 to 5.6% after the introduction of oral dextrose gel in clinical practice and, at last, even the retrospective study of Makker et al. [19] emphasized the significant impact of the oral treatment on the NICU admission rate and the number of IV dextrose administration. Similarly in our study the percentage of patients who required IV treatment was significantly reduced from 35.9 to 10% in the cohort of newborns managed with 40% oral dextrose gel. On the contrary, two recent studies did not demonstrate the efficacy of the dextrose gel to reduce NICU admission rates. In the former study [20], the Authors found a non-significant reduction from 2.5 to 1.5%; they tried to explain this result suggesting that the study took place in a Baby-Friendly Hospital with a low NICU admission rates already before the introduction of dextrose gel for the management of hypoglycemia. Ponnapakkam et al. [21] explained their controversial results by pointing out that despite a high compliance with dextrose gel usage, the skin-to-skin care and the early feeding are more comfortable measures for healthcare to prevent neonatal hypoglycemia.

The Cochrane review of 2016 [10] showed no significant differences in the need of IV treatment between the group of patients treated with oral dextrose gel and placebo group, but the Authors underlined the low quality of the two included studies due to inaccuracy of data collection, the presence of bias and deviations on the outcomes analyzed.

Our data showed that even the length of hospital stay of asymptomatic hypoglycemic newborns managed with dextrose oral gel in Gr2 was significantly shorter than newborns in Gr1. This result, of course, has to be evaluated considering the lower number of patients requiring NICU admission and the reduced need of IV treatment. In the same way, Rawat et al. [14] and Stewart et al. [22] showed a significant decrease of the length of hospital stay from 7.3 ± 4.3 to 3.1 ± 1.1 days and from 5.8 to 3.8 days, respectively. Conversely, Makker et al. [19] found no differences in the length of hospitalization between newborns managed with dextrose oral gel and IV treatment. Makker et al. [19] are aware of the study limitations: the comparison with a historical treated group with a prospective not controlled one, and the number of administered dextrose doses (4 doses), which is different in the respect of what proposed by other studies (6 doses).

Although in our study we did not directly perform a cost analysis, we observed the results of similar studies but we can assume, on the basis of literature [23], that a lower incidence of NICU admission, less IV treatment and a shorter length of hospitalization, make that oral dextrose gel is a less costly option for the management of neonatal asymptomatic hypoglycemia.

The rate of exclusive breastfeeding at discharge was the last secondary outcome evaluated in our study. Many data from the literature [24, 25] underline the crucial role of early initiation of breastfeeding and the skin-to-skin care immediately after birth. This is a crucial step to increase and promote maternal bonding and breastfeeding and to achieve successful exclusive breastfeeding during hospitalization and at discharge. Our results showed a significantly increased rate of newborns exclusively breastfed in Gr2: 30% (9/30) vs 7.7% (3/39) in Gr1. The possibility of not moving apart the mother-offspring dyad is attributable to the use of oral dextrose gel, easily administered in the maternity ward. As before reported, several others studies in literature confirmed our result, in particular the significant increase of exclusive breastfeeding in the newborns managed with oral dextrose gel [10, 14, 19]. On the other hand, two studies [20, 21] did not reach statistically significant increase in exclusive breastfeeding by the use of the oral dextrose gel, possibly related to the ongoing Baby-Friendly practices [20] and to the difficulties in healthcare staff organization [21].

To avoid these possible biases all the personnel dedicated to the application of the new protocol, physicians, nurses and residents of our ward, performed several and accurate training for the management of at-risk infants with oral dextrose gel. Thus, the administration of oral dextrose gel was well handled by the staff, well tolerated by newborns, and appreciated by the parents. Moreover, the administration of oral dextrose gel appears safe even in the long term, as indicated by the data in literature [26].

Furthermore, during our study did not occur any delay in breastfeeding establishment and duration as the literature may report when supplementation is used in neonatal period, thus as possible adverse effect of oral dextrose gel [27, 28]. However, our data show that newborns managed with oral dextrose gel present higher rate of exclusive breastfeeding.

Our study has several limitations: the study was only a comparison between a prospective group and a retrospective one managed with two different protocols for the management of asymptomatic hypoglycemia; there are some inhomogeneities between groups (see Table 1); the COVID 19 pandemic forced us to interrupt the Gr2 recruitment (due to less availability of hospital staff) and this group is less numerous; finally, we did not evaluate the rate of breastfeeding after discharge. This last data could be interesting to confirm the relevant role of dextrose gel to improve breastfeeding during the hospital stay in hypoglycemic infants.

## Conclusions

Future large randomized control trials are needed to provide further insight into the management with 40% oral dextrose gel for neonatal symptomatic hypoglycemia and to provide data about neurological outcomes. Once the effectiveness of the dextrose gel for the treatment of asymptomatic neonatal hypoglycemia has been confirmed, it would also be interesting to investigate, the role of this therapy in every single different risk factor for hypoglycemia, evaluating the short and long term outcomes.

## Data Availability

At V. Buzzi Children Hospital, Milan, Italy.

## Abbreviations

NICU: Neonatal intensive care unit
IV: Intravenous
LGA: Large for gestational age
IUGR: Intra uterine growth restriction
GA: Gestational age
SGA: Small for gestational age
AGA: Adequate for gestational age
LBW: Low birth weight
